# Identification of autism spectrum disorder based on functional near-infrared spectroscopy using adaptive spatiotemporal graph convolution network

**DOI:** 10.3389/fnins.2023.1132231

**Published:** 2023-03-10

**Authors:** Haoran Zhang, Lingyu Xu, Jie Yu, Jun Li, Jinhong Wang

**Affiliations:** ^1^School of Computer Engineering and Science, Shanghai University, Shanghai, China; ^2^Shanghai Institute for Advanced Communication and Data Science, Shanghai University, Shanghai, China; ^3^South China Academy of Advanced Optoelectronics, South China Normal University, Guangzhou, China; ^4^Key Lab for Behavioral Economic Science & Technology, South China Normal University, Guangzhou, China; ^5^Department of Medical Imaging Shanghai Mental Health Center, Shanghai Jiao Tong University School of Medicine, Shanghai, China

**Keywords:** autism spectrum disorder, functional near-infrared spectroscopy, multivariable time series, graph convolution network, adaptive spatiotemporal graph convolution network

## Abstract

The accurate diagnosis of autism spectrum disorder (ASD) is of great practical significance in clinical practice. The spontaneous hemodynamic fluctuations were collected by functional near-infrared spectroscopy (fNIRS) from the bilateral frontal and temporal cortices of typically developing (TD) children and children with ASD. Since traditional machine learning and deep learning methods cannot make full use of the potential spatial dependence between variable pairs, and require a long time series to diagnose ASD. Therefore, we use adaptive spatiotemporal graph convolution network (ASGCN) and short time series to classify ASD and TD. To capture spatial and temporal features of fNIRS multivariable time series without the pre-defined graph, we combined the improved adaptive graph convolution network (GCN) and gated recurrent units (GRU). We conducted a series of experiments on the fNIRS dataset, and found that only using 2.1 s short time series could achieve high precision classification, with an accuracy of 95.4%. This suggests that our approach may have the potential to detect pathological signals in autism patients within 2.1 s. In different brain regions, the left frontal lobe has the best classification effect, and the abnormalities occur more frequently in left hemisphere and frontal lobe region. Moreover, we also found that there were correlations between multiple channels, which had different degrees of influence on the classification of ASD. From this, we can also generalize to a wider range, there may be potential correlations between different brain regions. This may help to better understand the cortical mechanism of ASD.

## 1. Introduction

Autism spectrum disorder (ASD) is a serious brain disease with core symptoms: social communication impairment, verbal communication impairment, and repetitive stereotyped behaviors (Lord et al., 2000; Yang et al., 2019). Autism is more common in childhood (Blenner et al., 2011). In this spectrum disorder, childhood autism is one of the most serious childhood psychiatric disorders (Simonoff et al., 2008). Therefore, the early diagnosis and intervention treatment of ASD have great practical significance (Magán-Maganto et al., 2017).

Functional near-infrared spectroscopy (fNIRS) is widely used in cognitive neuroscience research (Pinti et al., 2020). Compared with other brain imaging techniques, this technique is insensitive to motion artifacts (Pinti et al., 2015). Therefore, fNIRS has unique advantages in targeting some special populations who cannot remain stationary for a long time, such as infants and ASD patients (Ehlis et al., 2014). The spontaneous hemodynamic fluctuation data from bilateral frontal and temporal cortices of children with ASD and TD collected by fNIRS are multivariate time series, with temporal and spatial correlations.

However, the current machine learning and deep learning methods cannot make full use of the latent spatial dependencies between pairs of variables, and require long time series to diagnose ASD. We collected relevant studies that also used the fNIRS dataset to diagnose ASD. For example, Xu et al. (2020a,b) adopted long time series of 1,000 units (70 s) and machine learning methods to classify ASD and TD. Xu et al. (2019) adopted long time series of 7 s and a deep neural network named CGRNN to diagnose ASD. The above studies have achieved good classification effect, but they all use single-channel fNIRS data, without the use of the spatial correlation of data, and use a long time series. This means that we need to collect more sample data to get more information.

Xu et al. (2021) adopted short time series of 3.5 s and a deep neural network named “CLAENTION” to diagnose ASD. They used multi-channel fNIRS data, but their classification accuracy was 86.3% on 44 channels data. They did not achieve better classification effect than the single-channel data. Because ordinary deep learning methods cannot deeply mine the latent spatial correlation between channels. Therefore, we consider modeling the fNIRS multivariate time series using graph convolution network. Graph networks have a better ability to capture spatial correlation, which can greatly shorten the length of time series. At the same time, the characteristics of graphs can also be used to directly mine the correlation between channels in the cerebral cortex. This provides new ideas for ASD medical research.

In recent years, research on autism spectrum disorders based on graph networks has also made some progress. For example, Yang et al. (2021) proposed a method named PSCR to construct a brain network graph, combined with graph attention network (GAT) to diagnose ASD, with an accuracy of 72.4%. Aslam et al. (2022) proposed a functional graph discriminative network (FGDN) to diagnose ASD on the basis of pre-defined graph. The above methods all need to diagnose ASD on the basis of pre-defined graph, which requires each researcher to have sufficient domain knowledge to construct graphs before modeling. Furthermore, the pre-defined graph cannot contain full spatial information about the data, nor are they directly related to the prediction task (Bai et al., 2020). Therefore, the above methods based on graph networks do not achieve good classification effect.

In summary, in this paper, we use an adaptive spatiotemporal graph convolution network (ASGCN) to classify ASD and TD without a pre-defined graph. We conduct a series of experiments on the fNIRS multivariate time series dataset. The main contributions of this paper can be summarized as follows:

(1) Only using 2.1 s short time series to achieve high precision classification of ASD and TD, since ASGCN model has strong ability to capture spatial features. This not only improves the efficiency of the data, but also suggests that our approach may have the potential to detect pathological signals in autism patients within 2.1 s.
(2) Through exploring the ability of different brain regions to identify ASD and TD, it is found that the left frontal lobe region performs the best classification, and the abnormalities occur more frequently in left hemisphere and frontal lobe region.
(3) By using the characteristics of graph, it is found that there are correlations between multiple channels, and it has different degrees of influence on the classification of ASD. And from this, we can generalize to a wider range, there may be potential correlations between different brain regions. This provides new perspective for ASD medical research.

## 2. Materials and methods

### 2.1. fNIRS data collection

In this study, the spontaneous hemodynamic fluctuations of each subject were recorded using a commercial continuous-wave fNIRS system (FOIRE-3000, Shimadzu Corporation, Kyoto, Japan). The subjects consisted of 25 ASD children (age 9.3 ± 1.4 years) and 22 TD children (age 9.5 ± 1.6 years). All ASD children were diagnosed by experienced clinicians in hospitals according to DSMIV-TR (American Psychiatric Association, 2013). In addition, it must be stated that before collecting fNIRS data, written consent was obtained from each subject and their parents, and the study protocol was approved by the Ethics Review Committee of South China Normal University (Zhu et al., 2014). It meets the Helsinki Declaration. During data collection, the subject's environment must be kept quiet and dim. Subjects closed their eyes and remained as still as possible.

**Figure 1:**
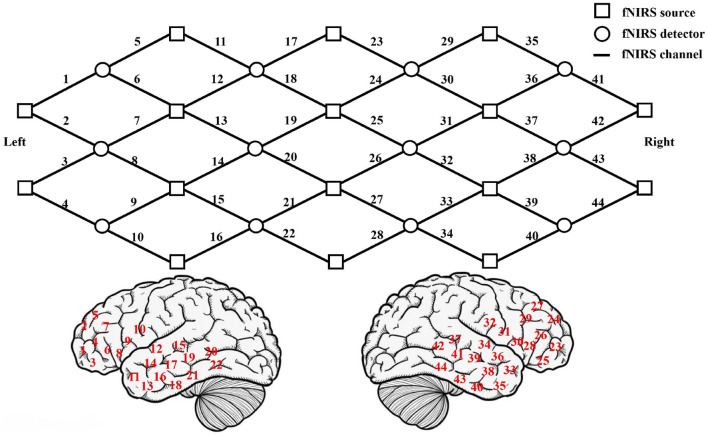
The **upper** picture shows the location of fNIRS measurement channels. The **lower** picture shows the location of each channel on the cerebral cortex.

The location of fNIRS measurement channels is showed in the front row of Figure 1. The squares represent the light sources and the circles represent the light source detectors. The lines between the squares and the circles represent the 44 channels of the brain, marked by 1-44. The location of each channel on the cerebral cortex (refer to the international 10-10EEG system to locate) is showed in the bottom of Figure 1. The left side is the location of the left hemisphere channel, and the right side is the location of the right hemisphere channel. The fNIRS detection areas include the left frontal lobe (channels 1–10), the left temporal lobe (channels 11–22), the right frontal lobe (channels 23–32), and the right temporal lobe (channels 33–44). The fNIRS uses the good scattering properties of the main components of blood to 600–900 nm near-infrared light to obtain the concentration changes of oxygenated hemoglobin (*HbO*_2_), deoxygenated hemoglobin (Hb) and total hemoglobin (HbT) during brain activity. The fNIRS light detector records the data of 44 channels of the cerebral cortex every 0.07 s. Each channel record contains 3 attributes (*HbO*_2_, Hb, and HbT concentration), and the total recording time is about 8 min.

### 2.2. Extraction and reconstruction of fNIRS short time series

Since we only have sample data of 47 subjects, if each subject is regarded as a sample, the amount of sample will be small, and it is very easy to overfit in the model training. Therefore, we use the method of sliding window (Feng and Jianhua, 2009) to traverse 47 sample data, and convert each sample data into a series of continuous and partially overlapping short time series. So as to increase the number of samples and realize the expansion of small sample data sets. Define the original fNIRS data as *D*, and each subject sample as *D*_*i*_(*i* ∈ [1, 47]) can be regarded as an *m* × *n* matrix, where *m* is the data length of the sample (the length of the sample data with a recording time of 8min is about 6857), and *n* is 132 (multiply the 44 channels and the 3 attributes of each channel).

**Figure 2:**
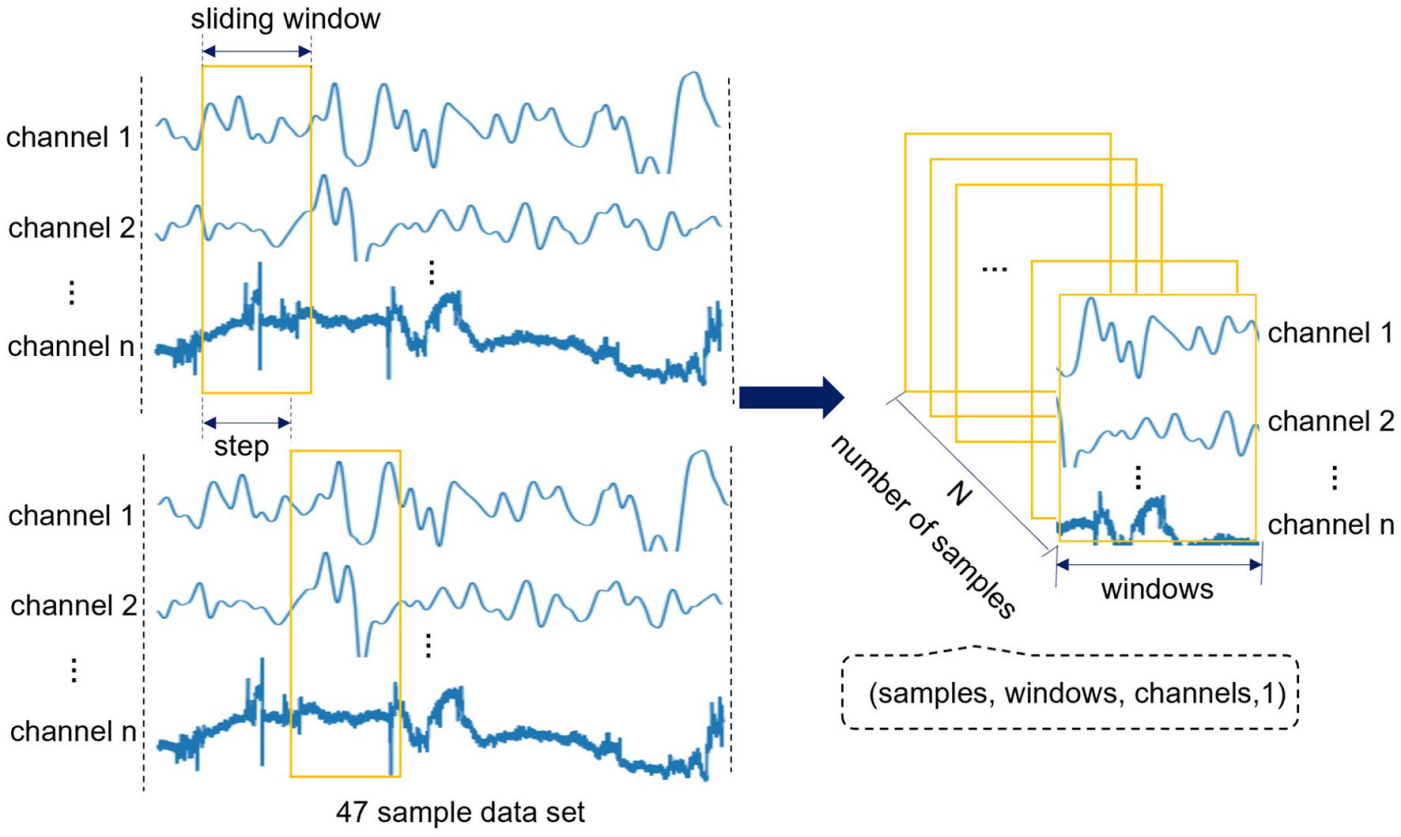
The specific process of fNIRS dataset extraction and reconstruction. This process sets the sliding window to traverse 47 sample data, and finally the format of original fNIRS data becomes four-dimensional tensor (samples, windows, channels, 1).

Suppose *w* represents the sliding window, *s* represents the time step (*s* < *w*), *c* represents the number of channels. Use sliding window to traverse each channel of each sample data *D*_*i*_ and select an attribute for each channel. One sample can get *N*_*i*_ = (*m* − *w*)/*s* subsequences, *N*_*i*_ is taken as an integer. Finally, 47 subject samples can get *N* = 47 × *N*_*i*_ subsequences. The specific process of fNIRS dataset extraction and reconstruction is shown in Figure 2. Finally, the format of original fNIRS data becomes four-dimensional tensor (samples, windows, channels, 1), i.e., (*N, w, c*, 1). In addition, add the corresponding label for each subsequence, 1 represents ASD children and 0 represents TD children. In this way, the format of the label becomes (*N*, 0*or* 1).

### 2.3. ASGCN model

**Figure 3:**
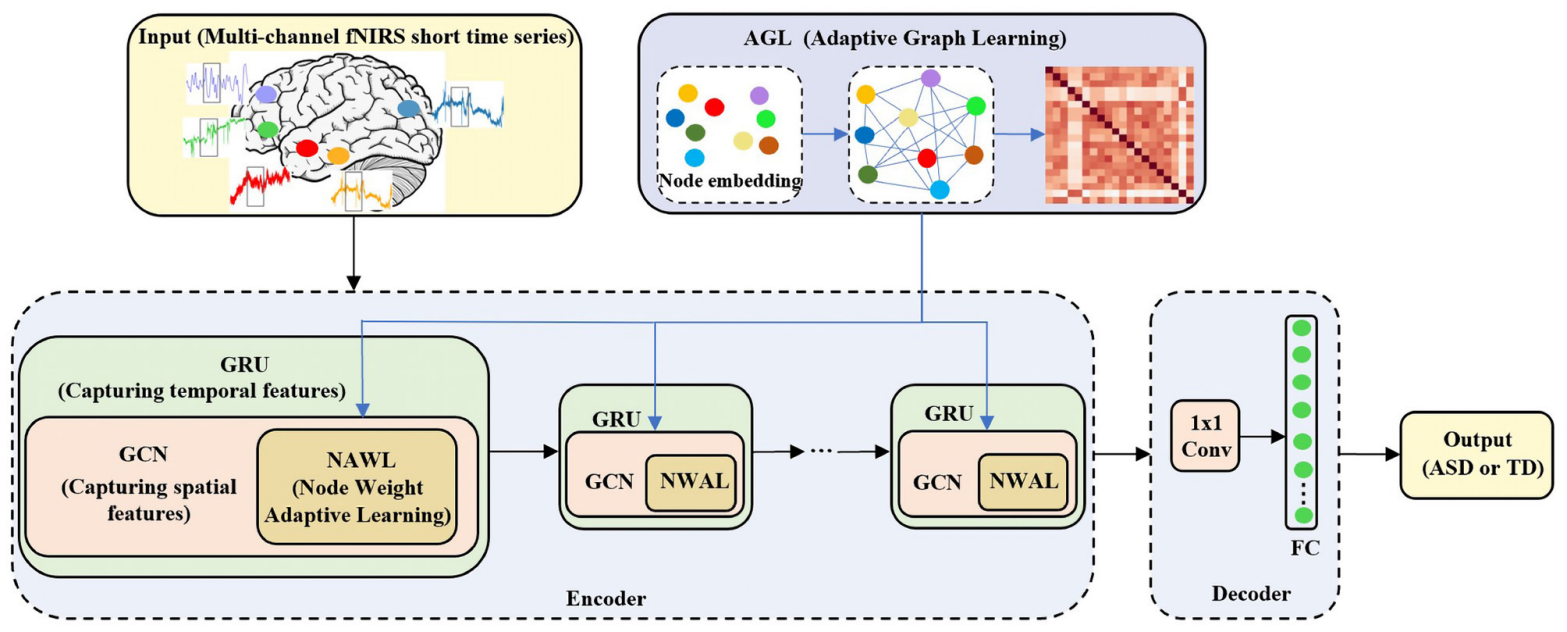
The specific structure of the ASGCN model. ASGCN model consists of an encoder and a decoder. The encoder part stacks multi-layer ASGCN modules, and each ASGCN module combines an improved GCN module (including adaptive graph learning (AGL) module and node weight adaptive learning (NWAL) module) and a GRU module. The decoder part consists of a 1 × 1 convolutional module and a fully connected layer.

In this section, we use the adaptive spatiotemporal graph convolution network (ASGCN) model to extract the features of the fNIRS data for classification. In order to use the characteristics of graphs to accurately capture the spatial correlation between channels in the cerebral cortex, the multi-channel fNIRS data in Section 2.2 is further formulated on the graph *G* = (*V, E, A*), where *V* represents the nodes of the graph, *E* represents the edges of the graph, and *A* represents the adjacency matrix of the graph and *A* ∈ *R*^*N*×*N*^. We regard each fNIRS channel as the node *V* and *V* = *N*, where N is both the number of nodes and the number of fNIRS channels. And we regard the correlation between fNIRS channels as the edge *E*. And *A* represents the graph adjacency matrix of the similarity between fNIRS channels. Therefore, the format of fNIRS data can also be defined as four-dimensional tensor (samples, time, nodes, feature), where time represents the size of sliding window, nodes represent the number of channels, feature represents the number of attributes and have a value of 1. This will also directly serve as input to the ASGCN model. The specific structure of the model is shown in Figure 3.

Our model stacks multi-layer ASGCN modules as an encoder. Each module consists of a combination of an improved graph convolution network (GCN) module and a gated recurrent unit (GRU) module. More specifically, there are two improvements in improved GCN module to better capture the spatial features of fNIRS data (e.g., the correlation between fNIRS multi-channel). The first is the adaptive graph learning (AGL) module. With AGL module, there is no need to predefine the graph structure. Instead, it automatically learns the graph adjacency matrix according to input data. Then the graph adjacency matrix is used as the input of each improved GCN module. The second improvement is the node weight adaptive learning (NWAL) module. In this module, the weights of the nodes can be adaptively learned to better capture spatial dependencies between nodes. Finally, it is combined with the GRU module to capture the temporal features of fNIRS data. The decoder part consists of a 1x1 convolutional module and a fully connected layer. It can transform our input channel dimension into the desired output (binary classification) dimension. Finally, output the result of classification, 1 (ASD) or 0 (TD).

#### 2.3.1. Extraction of spatial features of fNIRS multivariate time series

##### 2.3.1.1. Node weight adaptive learning module

GCN can learn the characteristics of nodes in the network and the information of network structure. In multivariate time series classification tasks, GCN can be used to capture the spatial correlation between variables (Wang et al., 2020; Wu et al., 2020). According to the calculation method proposed in the spectral domain (Defferrard et al., 2016; Kipf and Welling, 2016), GCN is calculated by using a good approximation of the first-order Chebyshev polynomial expansion as follows:


(1) $$\begin{array}{l} Z=\left(I_{N}+D^{-1/2}AD^{-1/2}\right)XW+b \end{array}$$

Where *A* ∈ *R*^*N*×*N*^ is the graph adjacency matrix, *D* is the degree matrix, *X* ∈ *R*^*N*×*C*^ and *Z* ∈ *R*^*N*×*F*^ are input and output of the GCN, *W* ∈ *R*^*C*×*F*^ and *b* ∈ *R*^*F*^ represent the learnable weights and bias. Furthermore, the computation can also be extended to higher dimensional GCN. This formula also expresses the main idea of GCN, which performs a weighted average of the neighbors of each node *X*^*i*^ ∈ *R*^1×*C*^ and its own information, so as to obtain a result vector *Z*^*i*^ ∈ *R*^1×*F*^ that can be transmitted to the network, and all nodes share *W* and *b*. But if weight parameters are assigned to each node, *W* ∈ *R*^*N*×*C*×*F*^ will become too large and cannot be optimized. In addition, it will bring serious overfitting problem. Therefore, we consider improving the traditional GCN model by a node weight adaptive learning (NWAL) module to solve this problem.

The weight parameter *W* ∈ *R*^*N*×*C*×*F*^ is not directly learned as the traditional GCN. In contrast, using the idea of matrix decomposition, it is generated by the shared weight pool $W_{m}\in R^{d\times C\times F}$ and the initialized node embedding matrix $E_{m}\in R^{N\times d}$, where *d* represents the embedding dimension of *E*_*m*_, *d*≪*N*. In other words, *W* is obtained by multiplying these two smaller parameter matrices, i.e., *W* = *W*_*m*_ · *E*_*m*_. Similarly, the bias parameter *b* can also be generated by the same way. In this way, the GCN improved by NWPL module does not need to assign weight parameters to each node, but can learn the node weight parameters adaptively. In summary, the calculation of GCN can be redefined as:


(2) $$\begin{array}{l} Z=\left(I_{N}+D^{-1/2}AD^{-1/2}\right)XE_{m}W_{m}+E_{m}b_{m} \end{array}$$

##### 2.3.1.2. Adaptive graph learning module

Existing GCN prediction models usually require a pre-defined graph for modeling. Pre-defined graphs are computed by distance functions or similarity measures (Geng et al., 2019). Therefore, the pre-defined graph cannot contain all the spatial information of nodes, and is not directly related to the classification task. Furthermore, predefining graphs is not an easy task without sufficient domain knowledge. And models based on GCNs on pre-defined graphs cannot be applied to other tasks.

Therefore, we make another improvement on the traditional GCN model. An adaptive graph learning (AGL) module is added to automatically learn the hidden spatial correlations between nodes from the data. First of all, the AGL module is randomly initialized to generate a learnable node embedding matrix $E_{A}\in R^{N\times d_{A}}$ for all nodes ($E_{A}^{T}$ represents its transpose matrix), where *d*_*A*_ represents the embedding dimension of nodes. Then multiplying *E*_*A*_ and $E_{A}^{T}$, passing through an activation function ReLU, and finally normalizing the adaptive graph adjacency matrix by the softmax function. This process can also be written as follows:


(3) $$\begin{array}{l} D^{-1/2}AD^{-1/2}=softmax\left(ReLU\left(E_{A}\cdot E_{A}^{T}\right)\right) \end{array}$$

These two matrices can be continually learned and updated during training, so as to learn the hidden spatial correlation between nodes (channels). It is worth noting that our final adaptive graph adjacency matrix is symmetric, because the graph adjacency matrix is obtained by multiplying *E*_*A*_ and $E_{A}^{T}$. Compared with the method of generating adaptive graph adjacency matrix in Wu et al. (2019), our method has better readability and interpretation. In summary, the calculation of GCN can be redefined as:


(4) $$\begin{array}{l} Z=\left(I_{N}+softmax\left(ReLU\left(E_{A}\cdot E_{A}^{T}\right)\right)\right)XW+b \end{array}$$

#### 2.3.2. Extraction of temporal features of fNIRS multivariate time series

In addition to spatial correlation, multivariate time series also have temporal correlation. We adopt gated recurrent unit (GRU) to capture the temporal dependencies of nodes (Fu et al., 2016). We use the improved GCN module (NWAL module and AGL module) to replace the MLP layer in GRU. The specific replacement process is as follows:


(5) $$\begin{array}{l} \tilde{A}=\;softmax\left(ReLU\left(E\cdot E^{T}\right)\right) \end{array}$$

(6) $$\begin{array}{l} z_{t}=\sigma \left(W_{z}\cdot \tilde{A}\left[h_{t-1},X_{t}\right]E+Eb_{z}\right) \end{array}$$

(7) $$\begin{array}{l} r_{t}=\sigma \left(W_{r}\cdot \tilde{A}\left[h_{t-1},X_{t}\right]E+Eb_{r}\right) \end{array}$$

(8) $$\begin{array}{l} {ĥ}_{t}=tanh\left(W_{ĥ}\cdot \tilde{A}\left[{r⊙h_{t-1},\;X}_{t}\right]E+Eb_{ĥ}\right) \end{array}$$

(9) $$\begin{array}{l} h_{t}=\left(1-z\right)⊙h_{t-1}+z⊙{ĥ}_{t} \end{array}$$

Where *W* and *b* represent the weight vector and the offset vector, *X*_*t*_ represents the value of input at time *t*, *h*_*t*_, and *h*_*t*−1_ represent the value of output at time *t* and *t* − 1, *z*_*t*_, and *r*_*t*_ represent the reset gate and the update gate, [ ] represents the concatenate operation of vectors. In addition, all node embedding matrices (*E*_*m*_ and *E*_*A*_) are all denoted by *E* and they are all learnable parameters. This will make sure that ASGCN model use a unified node embedding matrix, which can better explain our model.

## 3. Results

### 3.1. Experiments settings

In order to ensure the balance of positive and negative samples in the training set, validation set and test set, as well as the randomness of the samples, we first randomly shuffled 25 ASD children and 22 TD children respectively. Then we divide the two groups of children according to the ratio of 6:2:2, and then combine them. Ultimately, the training set contained 28 children (15 ASD and 13 TD), the validation set contained 10 children (5 ASD and 5 TD), and the test set contained 9 children (5 ASD and 4 TD). Then, we used sliding window of 30 units and time step of 10 units to segment, extract and reconstruct the data. Since the data is recorded once every 0.07s for a total of 8 min, the length of each short-time subsequence is 2.1 s, and the number of samples in the training set is expanded to about 19124. Finally, we convert the reconstructed data into a four-dimensional tensor (19124, 30, 44, 1) as the input of the ASGCN model, where 44 is the number of channels (or nodes), and 1 is the number of attributes (arbitrarily select one of *HbO*_2_, Hb, and HbT).

We set up to stack two layers of ASGCN modules. For the hyperparameters, the hidden units and the batch size are all set to 64 for all module units. For other parameters, we use the grid search method to select parameters with better experimental results, and finally set the learning rate to 0.001 and the node embedding dimension to 7. Besides, we choose binary cross-entropy loss as the loss function. During training, we did not use operations such as learning rate and weight decay. We use the Adam optimizer to iteratively train and optimize the model. The number of epochs is set to a maximum of 100. We also set an early stop strategy to prevent overfitting, i.e., the training stops when the value of patience on the validation set loss reaches 15. Our ASGCN model, and all other baseline comparison models, are implemented in Python using Pytorch 1.1.0, and executed on a computer with an Intel i5-10400F CPU and an Nvidia Geforce GTX 1660 Super GPU graphics card.

We use accuracy (Aghajani et al., 2017), sensitivity (Peng et al., 2016), specificity (Gateau et al., 2015), precision, and AUC (area under the curve) as performance evaluation metrics for classification models, which are commonly used in the medical field. Accuracy is the percentage of all children diagnosed correctly. Sensitivity refers to the probability that an actual ASD patient will be correctly judged as positive (true positive). Specificity refers to the probability that an actual TD child will be correctly judged negative (true negative). Precision refers to the proportion of correct ASD predictions to total ASD predictions. AUC (area under the curve) is a criterion for judging the performance of a classifier. Let TP denote the number of actual ASD and predicted ASD. TN represents the number of actual TD and predicted TD. FN represents the number of actual ASD but predicted TD. FP represents the number of actual TD but predicted ASD. Thus, These formulas for these evaluation metrics can be expressed as follows:


(10) $$\begin{array}{l} Accuracy=TP+FN/\left(TP+FP+FN+TN\right) \end{array}$$

(11) $$\begin{array}{l} Sensitivity=TP/\left(TP+FN\right) \end{array}$$

(12) $$\begin{array}{l} Specificity=TN/\left(TN+FP\right) \end{array}$$

(13) $$\begin{array}{l} Precision=TP/\left(TP+FP\right) \end{array}$$

### 3.2. Setting of short time series

In order to achieve high precision classification and shorten the required time series length and training time as much as possible, we set three groups of experiments with sliding window length of 20, 30, and 50, and time step is set to 10. Since the experimental data is recorded every 0.07 s, different sliding window length can be defined as short time series of 1.4, 2.1, and 3.5 s respectively. Besides, other parameters are set the same, select 44 channels as the number of nodes (that is, use the data of the entire brain region), and select *HbO*_2_ as the attribute. In this section, we add training time as an evaluation metric. Due to the early stop strategy, the number of training times for each experiment is not fixed. So the average training time of a single epoch is used as the evaluation metric. The experimental results are shown in Table 1.

**Table 1:** Comparison of experimental results for different sliding window lengths.

**Sliding window**	**Accuracy**	**Precision**	**Sensitivity**	**Specificity**	**AUC**	**Training time (min/epoch)**
1.4 s	0.859	0.894	0.846	0.839	0.861	1.584
2.1 s	0.954	0.974	0.937	0.972	0.955	2.640
3.5 s	0.926	0.929	0.896	0.947	0.905	4.568

It can be seen from Table 1 that the classification accuracy of the three groups of sliding window are all above 85%, which shows that our model has certain classification ability for ASD and TD. Although the training time of 1.4 s is the shortest, the classification effect is not the best. This shows that the short time series of 1.4 s does not contain enough feature information for classification. The classification effect of 3.5 s is better than that of 1.4 s, but the training time is the longest. The classification effect of 2.1 s is the best (the highest accuracy, 95.4%), and the training time is not long. This shows that the short time series of 2.1 s can contain enough feature information for classification. In summary, we believe that the ASGCN model can extract the enough spatial feature information of fNIRS multivariate time series, thereby shortening the required time series length. This also provides the basis for our subsequent experiments.

### 3.3. Selection of fNIRS attributes

Since the HbT (HbT=*HbO*_2_+Hb) in the fNIRS data is not an independent attribute, this experiment only uses two attributes of *HbO*_2_ and Hb as the experimental data for comparison. We still set 2.1 s as sliding window and use the data of the entire brain region (44 channels). Select an attribute with better classification effect on ASD to provide a basis for subsequent experiments. The experimental results are shown in Table 2.

**Table 2:** Comparison of experimental results for different attributes.

**Attribute**	**Accuracy**	**Precision**	**Sensitivity**	**Specificity**	**AUC**
*HbO*_2_	0.954	0.974	0.937	0.972	0.955
*Hb*	0.897	0.902	0.902	0.892	0.897

It can be seen from Table 2 that the classification effect of the two attributes is good (evaluation metrics are all above 89%), but the classification effect of the *HbO*_2_ attribute is better. Therefore, the subsequent experiments all use the *HbO*_2_ attribute as the experimental data.

### 3.4. Classification effect of different brain regions

In Section 3.2, we use the entire brain region (44 channels) as experimental data, and obtain a good classification effect. In order to explore the influence of different brain regions on the classification effect of ASD, we further divide the brain regions into: left hemisphere (channels 1–22), right hemisphere (channels 23–44), left frontal lobe region (channels 1–10), left temporal lobe region (channels 11–22), right frontal lobe region (channels 23–32), and right temporal lobe region (channels 33–44). The six regions are taken as experimental objects to explore the influence of different brain regions on the classification of ASD. We still choose 2.1 s as sliding window and *HbO*_2_ as attribute. Only accuracy, sensitivity and specificity are selected as evaluation metrics. The experimental results are shown in Table 3.

**Table 3:** Comparison of classification effect in different brain regions.

**Brain region**	**Accuracy**	**Sensitivity**	**Specificity**
Left hemisphere	0.902	0.962	0.842
Right hemisphere	0.865	0.759	0.983
Left frontal lobe	0.959	0.953	0.966
Left temporal lobe	0.937	0.914	0.963
Right frontal lobe	0.909	0.827	0.998
Right temporal lobe	0.834	0.717	0.963

Accuracy represents the ability of the model to distinguish ASD from TD, sensitivity represents the ability of the model to diagnose ASD, and specificity represents the ability of the model to diagnose TD. First, it is obvious that the left frontal region lobe has the highest classification accuracy of 95.9%. This shows that the left frontal lobe region has the best classification effect and the strongest ability to distinguish ASD and TD. Second, for sensitivity, left hemisphere is significantly higher than right hemisphere, left frontal lobe and left temporal lobe regions are also higher than right frontal lobe and right temporal lobe regions. This shows that the left hemisphere is more capable of diagnosing ASD and more sensitive to ASD. In contrast, for specificity, right hemisphere is higher than left hemisphere. This shows that the right hemisphere is more capable of diagnosing TD and more sensitive to TD. In addition, the sensitivity of the left frontal lobe was higher than the left temporal lobe, and the sensitivity of the right frontal lobe was higher than the right temporal lobe. This shows that the frontal lobe region is more capable of diagnosing ASD and more sensitive to ASD. In summary, This reflects the abnormalities occurred more frequently in left hemisphere and frontal lobe region. And if only the most rapid diagnosis of ASD is desired, the fNIRS signal can be used to acquire data from the left frontal region with best classification performance.

### 3.5. Multi-channel correlation mining and its influence on ASD classification

**Figure 4:**
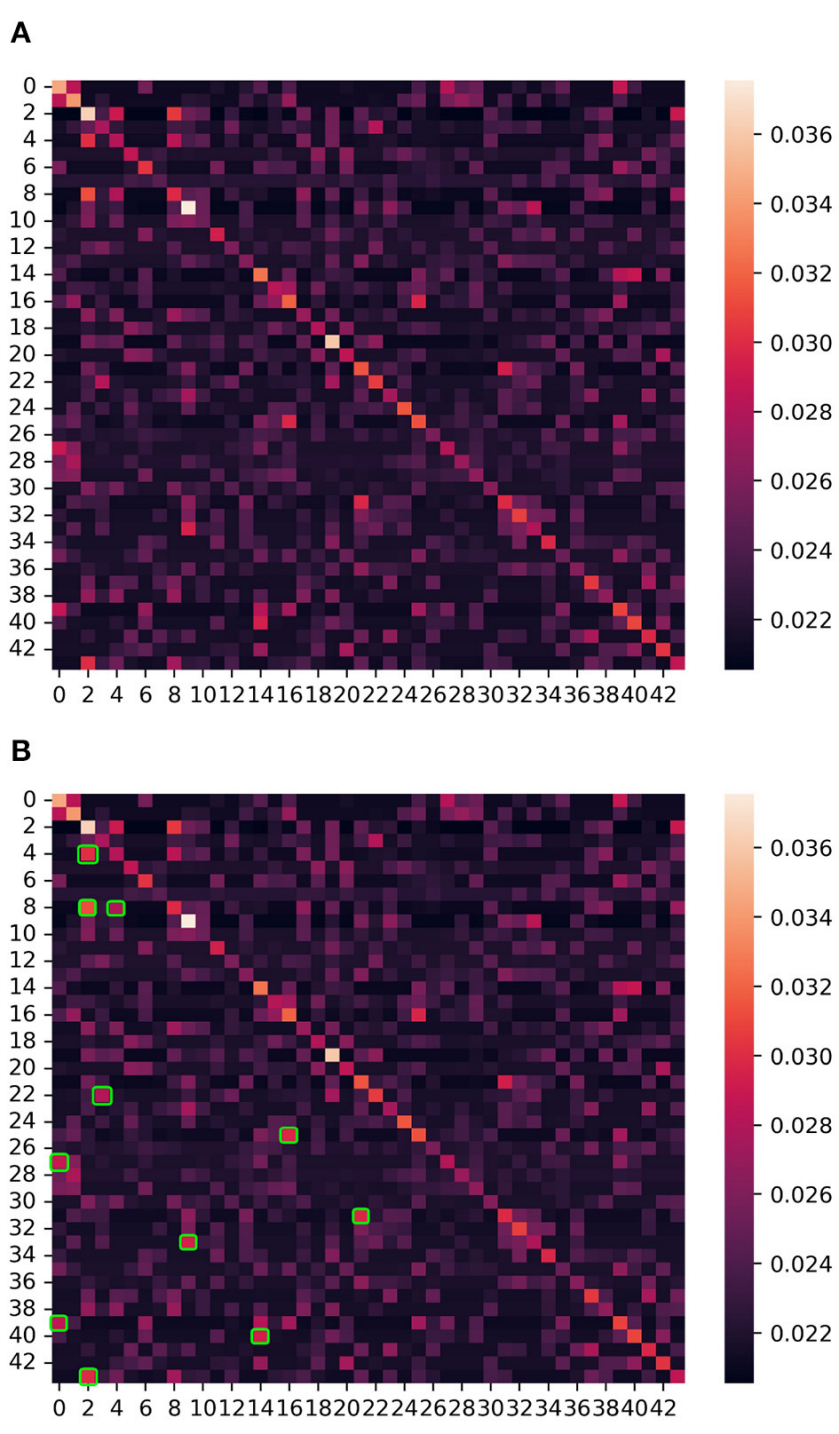
The heat map of the graph adjacency matrix of the entire brain region (i.e., 44 channels). The numbers 0–43 on the left and below represent channels 1–44, and one channel corresponds to one node of the graph. Each small square on the figure represents the correlation (i.e., edge) between channels, and the color of the square represents the degree of correlation between channels (i.e., weight on the edge). The correlation measurement standard is shown in the color axis and number axis on the right. The brighter the color and the larger the value, the stronger the correlation between two channels (nodes). In addition, the main diagonal represents the autocorrelation of these channels (nodes), and the regional correlations on both sides of the main diagonal are completely symmetrical. **(A)** The heat map of the entire brain region. **(B)** The strong correlation (bright areas) regions are marked with green squares.

In this section, we use the graph adjacency matrix generated by the ASGCN model to directly mine the correlation between channels, and analyze the influence of the correlation on the ASD classification effect. The heat map of the graph adjacency matrix of the entire brain region (44 channels) is shown in Figure 4A. We conduct experiments based on this figure. It is noted that the graph adjacency matrix is symmetric (the reason is explained in Section 2.3.1.2).

**Figure 5:**
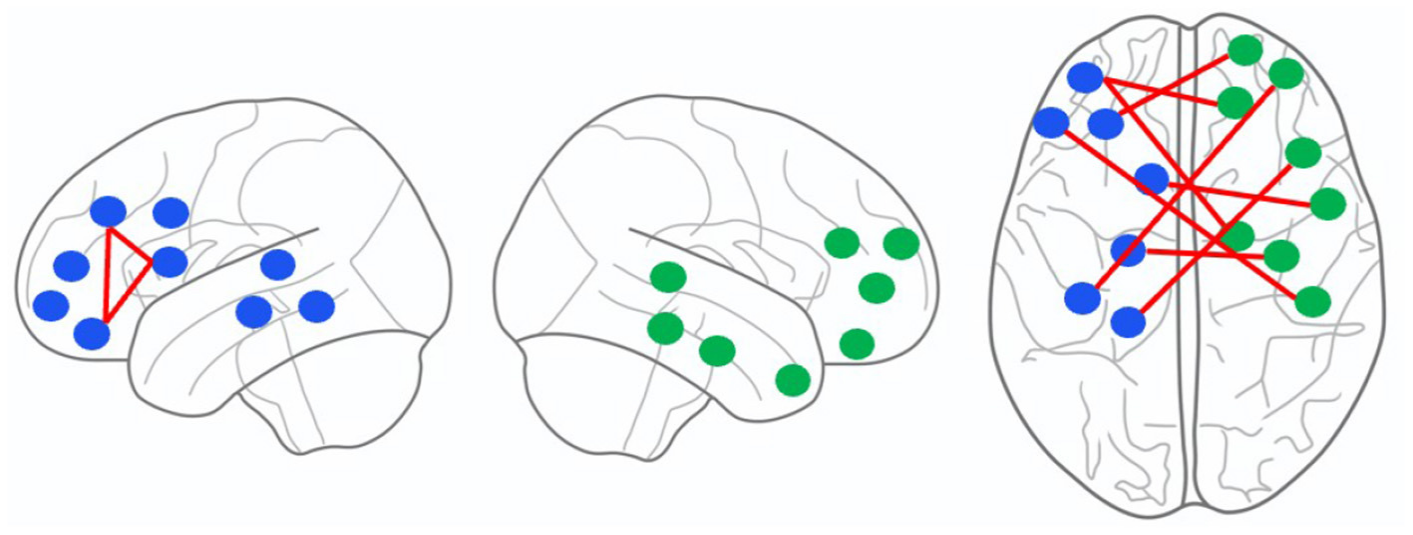
The location of channels with strong correlation on the cerebral cortex. From **left** to **right**, it is shown as the left hemisphere, the right hemisphere and the whole cerebral cortex. The blue and green dots represent channels, and the red lines represent the edges between channels.

The channel pairs with strong correlation are marked in Figure 4B, as follows: channel 3 and 5, channel 3 and 9, channel 5 and 9, channel 4 and 23, channel 17 and 26, channel 1 and 28, channel 22 and 32, channel 10 and 34, channel 1 and 40, channel 15 and 41, and channel 3 and 44. First, we project the location of this above channels with strong correlation on the cerebral cortex, as shown in Figure 5. It can be seen that the channels with strong correlation are mostly located in the left frontal lobe region or between the left and right hemispheres. And there are no strong correlation channels in the right hemisphere. First, this is consistent with our experimental conclusion in Section 3.4 (the model has best classification effect in left frontal lobe region), because more potential correlations between channels can be mined in left frontal lobe region. Furthermore, there are correlations in many channels between the left and right hemispheres. This shows that there may be potential correlation between the left and right hemispheres.

**Figure 6:**
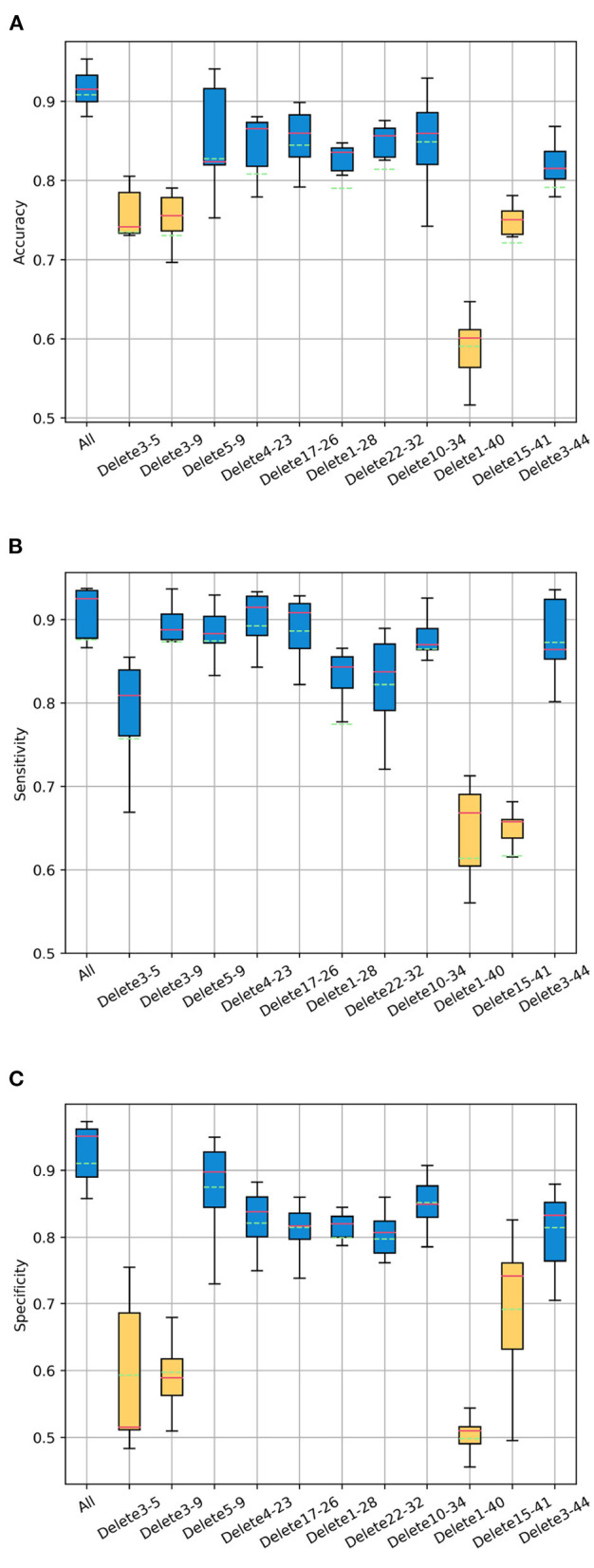
The changes of the ASD classification effect after deleting the edges between channel pairs. Boxplots represent the variant of classification **(A)** accuracy, **(B)** sensitivity, and **(C)** specificity of test sets in multiple epochs during training. The abscissa “All” represents no edges are deleted. The abscissa “Delete 3–5” represents deleting the edges between channel 3 and channel 5, and so on.

Next, we conduct an experiment on the influence of correlation on ASD classification. The experiment takes the classification effect of the whole brain region (44 channels) as the baseline, still chooses 2.1 s as sliding window and *HbO*_2_ as the attribute. Accuracy, sensitivity and specificity are selected as evaluation metrics. Deleting the edges between above channel pairs respectively (i.e., the value of correlation between channel pairs is set to 0), and then observing the changes of the ASD classification effect. More generally speaking, the bright areas (include the areas symmetrical to the main diagonal) marked in Figure 4B are all set to be dark, other areas remain unchanged. At the level of the graph neural network, that is, deleting the edges between nodes and keeping the nodes. The experimental results are shown in Figure 6.

For the accuracy, the relatively large decline is to delete the edges between channel 1 and 40, channel 3 and 5, channel 3 and 9, channel 15 and 41. For the sensitivity, the relatively large decline is to delete the edges between channel 1 and 40, channel 15 and 41. For the specificity, the relatively large decline is to delete the edges between channel 1 and 40, channel 3 and 5, channel 3 and 9, channel 15 and channel 41. This shows that the relatively great influences on the ASD classification effect are as follows: the correlation between channel 1 and 40, channel 3 and 5, channel 3 and 9, channel 15 and 41.

### 3.6. Identification ability comparison of different classification models

**Figure 7:**
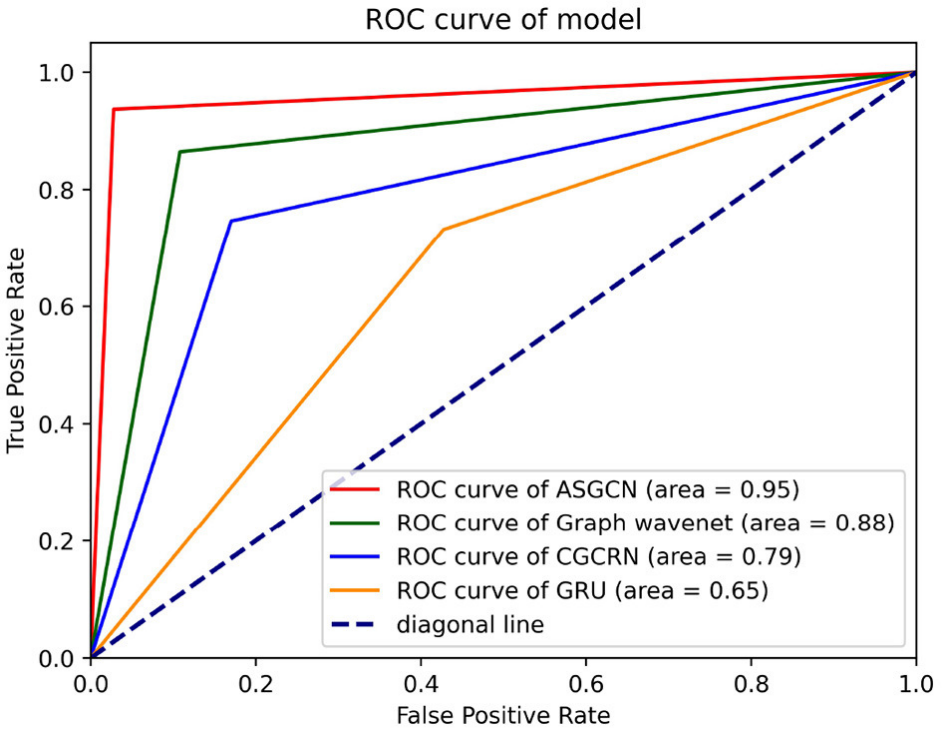
ROC curve of different classification model. Each test selected three thresholds, i.e., max value, min value, and mid-value. Each threshold corresponded to a point (false positive rate, true positive rate). All coordinate points were connected to draw the ROC curve. The area represents AUC.

The purpose of this experiment is to evaluate the performance of the ASGCN model, compared with GRU (Dutta, 2019), CGCRN (Xu et al., 2019), Graph wavenet (Wu et al., 2019) models. Each experiment used the fNIRS data of whole brain region as the input of model. We still set the sliding window to 2.1 s and attribute to *HbO*_2_. ROC curve and AUC (area under the curve) are selected as evaluation metrics. The higher the AUC value and the closer the ROC curve is to the top left corner(0, 1), the better the classification performance of the model. The experimental results are shown in Figure 7.

The AUC value of our model reaches the highest 0.95, and the ROC curve is closest to the top left corner (0, 1), so it has the best classification effect. This shows that our model has a strong ability to capture temporal and spatial features. The second is the Graph Wavenet model, which is an adaptive spatiotemporal graph network model similar to our model. This model also has a certain ability to capture temporal and spatial features. But based on the part of this model, the AGL module in our model has made some improvements. Therefore, our model classification performance is slightly better than this model. The third is the CGCRN model, a combination of deep neural network models, which is relatively weak in capturing spatial features. The worst is the GRU model, which basically has no ability to capture spatial features. In all, this demonstrates the superiority of our model in capturing the temporal and spatial features of multivariate time series. And our model does not need to pre-define the spatial graph structure, which means that researchers do not need to spend a lot of time learning enough domain knowledge in advance.

## 4. Discussion

In this study, we use the method of sliding window to divide the original fNIRS long time series (8 min) into multiple overlapping short time series (2.1 s). First, this complete the expansion of the original small-sample dataset. Next, since ASGCN has a strong ability to capture spatial features, the fNIRS multi-channel data is used to increase the spatial feature information required for classification, thereby shortening the required time series length. We achieve high precision classification of ASD and TD using ASGCN model and 2.1 s short time series. Compared with the length of the time series (3.5 s) in Xu et al. (2021), the required length of our model is reduced by 40%. This not only improves the efficiency of data and solve the problem of insufficient medical data, but also suggests that our approach may have the potential to detect pathological signals in autism patients within 2.1 s.

We further analyze the channels with greater influence on ASD classification effect obtained in section 3.5 experiment conclusion, including channel 1, channel 3, channel 5, channel 9, channel 15, channel 40, and channel 41. We redistrict the cerebral cortex using the Brodmann partition system (Šimic and Hof, 2015). Channel 1 and 3 are located in area 11 of Brodmann area, which are located in the prefrontal lobe cortex. This area has advanced cognitive functions and is responsible for all aspects of thinking, judgment and perception, memory and recall of information, problem solving, emotions, etc. Channel 5 is located in area 10 of Brodmann area, which is located in the medullary area on top of the inferior frontal gyrus. Brodmann areas 10 and 11 together make up the function of the prefrontal lobe cortex. Damage to these two areas can lead to cognitive impairment, including social and emotional impairment (Lake et al., 2019), as well as symptoms such as paranoid personality, narrowed interests, and intellectual disability. These symptoms are also some of the core symptoms of autism patient (social communication impairment and repetitive stereotyped behaviors). Channel 9 is located in area 44 of Brodmann area, which is located in the triangular area of the inferior frontal gyrus. This area is also called Broca's area, motility language area, is related to the production of language and performs semantic tasks (Gaffrey et al., 2007). Broca's area damage can lead to expressive aphasia, that is only can use simple and limited vocabulary repeatedly, but not use complex grammar and syntax. They are able to understand language, but they are unable to respond (Islam et al., 2022). Channels 15, 40, and 41 are located in area 22 of Brodmann area, which are located in the superior temporal gyrus. Its anterior part belongs to Wernicke's area. Wernicke's area is the auditory and visual language center of the brain, responsible for language comprehension. Damage to Wernicke's area can lead to severe sensory aphasia and loss of auditory memory (Hitoglou et al., 2010; Zhang et al., 2022). Patients can hear voices, speak to themselves, but cannot understand what others are saying. Severe cases cannot even distinguish human saying from other types of sounds. The symptoms of damage to Broca's area and Wernicke's area together make up to another core symptom of autism (verbal communication impairment).

In summary, it can be found that these channels with strong correlation and great influence on ASD classification are located in some areas highly related to core symptoms of autism. First, this reflects the strong ability of our model to capture spatial features, which can give higher weight to the edges between channels with stronger correlation. Second, from the correlations between these channels, we can generalize to a wider range, that is, there may be potential correlations between different brain regions. For example, there may be a potential correlation between Brodmann's area 11 and area 22 or the prefrontal cortex and superior temporal gyrus. Our future work will also focus on the above inferences, remodeling the fNIRS data through ASGCN model, directly obtaining the spatial connection graph structure between different brain regions, and mining the potential correlation between different brain regions.

## 5. Limitations and conclusion

First, we would like to state that this study has some limitations. Although we extended the original small sample by setting a sliding window, due to the small number of original samples (only 47 subjects), the extrapolation ability of the model may have certain limitations, which may affect the generalizability of the model. Therefore, further studies are warranted, for example, applying ASGCN model to other multivariate time series classification tasks, to verify and improve the model's generalizability.

In this paper, we classify ASD and TD using adaptive spatiotemporal graph convolution network model. This method does not require a pre-defined graph, and can automatically learn to generate graphs through data. We first expand the fNIRS multivariate time series data using sliding window and reconstruct it into the input format of the model. Using the powerful spatial feature capture ability of the graph convolution network, we can achieve good classification effect with only a short time series of 2.1 s. Second, we conduct classification experiments in different brain regions. The results shows that the left frontal lobe region performs best for classification, and the abnormalities occur more frequently in left hemisphere and frontal lobe region. Moreover, we use the graph generated by ASGCN model to directly mine the correlation between channels and its influence on ASD classification, so as to explore the potential correlation inside the cerebral cortex. This provides a new perspective for ASD medical research. Finally, we use ROC curve to compare our model with other models to demonstrate the superiority of our model.

## Data availability statement

The raw data supporting the conclusions of this article will be made available by the authors, without undue reservation.

## Ethics statement

The studies involving human participants were reviewed and approved by Ethics Review Committee of South China Normal University. Written informed consent to participate in this study was provided by the participants' legal guardian/next of kin.

## Author contributions

HZ conducted the experiments and wrote the manuscript. LX offered important help on guiding the experiments and methods. JY reviewed and revised the manuscript. JL and JW analyzed the feasibility of the article from a perspective of fNIRS brain imaging and revisited the manuscript critically. All authors contributed to the article and approved the submitted version.
